# Suicidal behaviour, depression and generalized anxiety and associated factors among female and male adolescents in Mozambique in 2022–23

**DOI:** 10.1186/s13034-024-00835-8

**Published:** 2024-11-07

**Authors:** Supa Pengpid, Karl Peltzer, Boia Efraime

**Affiliations:** 1 Department of Health Education and Behavioral Sciences, Faculty of Public Health, Mahidol University, 420/1 Ratchawithi Road, Ratchathewi, Bangkok, 10400 Thailand; 2 Department of Public Health, Sefako Makgatho Health Sciences University, Pretoria, South Africa; 3 Department of Healthcare Administration, College of Medical and Health Science, Asia University, Taichung, Taiwan; 4 Department of Psychology, University of the Free State, Bloemfontein, South Africa; 5 Department of Psychology, College of Medical and Health Science, Asia University, Taichung, Taiwan; 6 Associação de Psicologia de Moçambique, Maputo, Mozambique; 7 Associação Reconstruindo a Esperança, Maputo, Mozambique

**Keywords:** Adolescents, Suicidal behaviour, Generalized anxiety disorder, Major depressive disorder, National survey, Mozambique

## Abstract

**Background:**

The purpose of the study was to assess the prevalence and associated factors of major depressive disorder (MDD), generalized anxiety disorder (GAD), and past 12-month suicidal behaviour (PSB) among adolescents in Mozambique.

**Methods:**

Data from 3,109 females (aged 15–19 years) and 1,439 males (aged 15–19 years) that participated in the 2022-23 Mozambique Demographic and health Survey were analysed. MDD was assessed with the PHQ-9 and GAD with the GAD-7.

**Results:**

Results indicate that among girls the prevalence of PSB was 4.3% (attempt 1.0%, plan 1.9% and/or ideation 3.6%) and among boys 2.5% (attempt 0.3%, plan 0.7% and/or ideation 2.4%). Among girls and boys, the prevalence of MDD (≥ 8 scores) was 15.5% and 3.7%, respectively, and the prevalence of GAD (≥ 5 scores) was 25.0% and 10.3%, respectively. In adjusted logistic regression analysis, among girls, GAD was positively and solid fuel use was negatively associated with PSB, while among boys MDD and urban residence were positively associated with PSB. Among female adolescents, currently being pregnant and “big problem to get money for medical treatment” increased the odds of MDD. While among male adolescents, urban residence, having a genital sore or ulcer, has living children, and early sexual debut were positively associated with MDD. Urban residence, current alcohol use, and early sexual debut were positively associated with GAD in male adolescents, while poorer wealth status, being pregnant, and having a “big problem to get money for medical treatment” were positively associated with GAD in female adolescents.

**Conclusion:**

About 3% of participants had PSB, among girls one in five had MDD or GAD and among boys more than 5% had MDD or GAD. Public health interventions can be guided by several associated factors that have been identified.

## Background

For adolescents aged 15 to 19 years, suicide ranks as the fourth most common cause of death [1]. A significant risk factor for successful suicide among young people is having attempted suicide [2]. Preventing suicide risk can be achieved by identifying and tracking the prevalence of suicidal behaviour and its correlates [3]. On the other hand, there is a paucity of information regarding suicidal behaviour among adolescents in Mozambique’s general population. Results from a national adolescent school survey in Mozambique show that 18.0% (20.0% among girls 17.6% among boys and) attempted suicide [4] and 17.7% reported suicidal ideation in the past 12 months [5]. In a local survey of adolescents (ages 12–19 years) in two schools, the prevalence of past-month suicidal ideation and planning was 16.4% and 11.3%, respectively [6]. According to Liu et al. [7], 17.2% of school-age adolescents in 40 low- and middle-income countries (LMIC) reported having attempted suicide in the past 12 months. Nyundo et al. [8] studied adolescents at eight community sites in six sub-Saharan African nations, and the prevalence of past 12-month suicidal behaviour (ideation, plan, and/or attempt) (PSB) ranged from 1.2 to 12.4% in the eight sites.

Worldwide, it is projected that 14% of teenagers (10–19 years old) suffer from a mental illness (primarily anxiety, depression, and behavioural disorders), accounting for 13% of the adolescent disease burden [1]. According to Tinsae et al. [9], there was a 27.4% overall prevalence of mental health distress among adolescents in Africa. Generalized anxiety disorder (GAD) and major depressive disorder (MDD) are common mental disorders that often co-occur [10]. There is a dearth of national community-based data on GAD and MDD among adolescents in Mozambique. Among 492 school adolescents in Maputo, Mozambique, 33.3% met criteria for any mental health diagnosis based on the Mini-International Neuropsychiatric Interview for Children and Adolescents (MINI-KID), most commonly anxiety disorder (17.7%), and major depressive episode (8.5%) [11]. In sub-Saharan adolescents the general population prevalences were 26.9% for depression, and 29.8% for anxiety disorders [12]. Of the youth and adolescents (10–24 years old) who sought out psychological services at eight “Servicios Amigos dos Adolescentes” in the Sofela Province of Mozambique (50% of the sample tested positive for HIV), 9% had GAD-7 (≥ 10 scores), and 7.3% had PHQ-9 (≥ 11 scores) [13]. In two local studies among adolescents in South Africa, one in Khayelitsha, Cape Town, 32.1% had MDD (PHQ-9: ≥10 scores) and 17.8% had GAD (GAD-7: ≥10 scores) [14], and one in the Western Cape, 33.5% had depression (PHQ-A: ≥8 scores) and 20.9% anxiety (GAD-7: ≥6 scores) [15].

Factors associated with suicidal behaviour in adolescents may include psychosocial distress [7, 16, 17], and depressive symptoms [8, 18], sociodemographic factors, including older age [7], lower socioeconomic status [19], female sex [7]; and social environmental factors, including substance use [4], lack of social support [16], sexual risk behaviour [20], and poor access to health care [8].

Socio-demographic factors associated with MDDs among adolescents may include female sex [8, 21], older adolescent age, and greater wealth status [22]. The history of a sexually transmitted infection (STI) [23], genital sores or ulcers, and low self-rated health status [22] are health status variables linked to MDD in adolescents. Poor diet, such as a lack of variety in foods consumed [24], substance use, such as smoking cigarettes [25], alcohol use [22, 26], early sexual debut [22, 27], and sexual risk behaviour [28] are health risk behaviours linked to MDDs in adolescents. Pregnancy loss [22, 29], being pregnant [22, 30], and using solid biomass fuel in the home [31] are stressors linked to MDDs in adolescents.

Adolescent GADs may be related to sociodemographic factors such as female sex [13], younger age [25], and higher wealth status [22]. Poor self-rated health status [22, 32], pregnancy loss [22], alcohol use [22, 26], perceived low social support [25], HIV positive status [13, 33] and inadequate medical care accessibility [22] are some of the health statuses, health risk behaviours, and stressors linked to GADs in adolescents.

In Mozambique in 2022–2023, the study sought to assess the prevalence and correlates of GAD, MDD, and PSB in male and female adolescents. The findings may contribute to bettering Mozambique’s mental health policy and the provision of services for adolescents with mental health issues.

## Methods

The sample was limited to individuals who completed the mental health module, and we included teenagers (15–19 years old) in the Mozambique Demographic and Health Survey (MDHS) for 2022–2023. A household-based sample that was nationally representative was included using a multi-stage sampling design; the response rate for the women’s and men’s interview sample was 97% and 94%, respectively [34]. The 2022 NDHS was approved by the “Instituto Nacional de Estatística (INE) and ICF Institutional Review Board,” and written informed consent was obtained from the household head, and a parent or guardian must provide consent prior to participation by a child or adolescent to conduct interviews [34].

### Measures

#### Outcome variables

Suicidal behaviour questions included PSB (attempt, plan, and ideation): “In the past 12 months have you seriously considered a suicide attempt? (Yes/No)” “In the past 12 months, have you made a plan for how you would attempt suicide? (Yes/No)” “In the past 12 months, have you made a suicide attempt? (Yes/No).” [34].

The Patient Health Questionnaire (PHQ-9)’s nine items on common depressive symptoms over the previous two weeks were used to evaluate MDD [35]. Responses were rated from 0 ‘not at all’ to 3 ‘always’. The PHQ-A found that adolescents in Mozambique [36] had an ideal cutoff score of 8 and showed good sensitivity and specificity (> 0.70). Cronbach alpha of the PHQ-9 was 0.87 among females and 0.79 among males in this sample.

The seven-item (GAD-7) scale was used to measure GAD [37]. The GAD-7 showed good sensitivity and specificity (> 0.70) and found that among adolescents in Mozambique [36], a cutoff score of 5 was ideal for moderate or severe GAD. Cronbach alpha of the GAD-7 was 0.91 among females and 0.80 among males in this sample.

*Mental health care* utilization included, (1) “During the last 2 weeks, did you take medicine prescribed by a doctor or other healthcare worker for depression or anxiety?” (2) “During the last 2 weeks, did you take medicine prescribed by a doctor or other healthcare worker for any other mental health condition?”

For those who had experienced any symptoms from the GAD-7 and/or PHQ-9 in the past two weeks were asked: “Thinking about what you yourself have experienced among the different things we have been talking about, have you ever tried to seek help?” Response options were (1) doctor/medical personnel, (2) social service organization, (3) social worker, (4) community health worker/field worker, (5) religious leader, (6) current/former spouse/partner, (7) other family member, (8) friend, (9) neighbour, (10) Other, specify… [34].

Sociodemographic factors assessed included age, marital status, education, wealth status, work status and geolocality (rural, urban).

## Health status and behaviour

*Self-rated health status* was classified as 1 = bad or very bad and 0 = very good, good, or moderate. History of sexually transmitted infection (STI) in the past 12 months, and ever tested positive for HIV. For males, *genital sore or ulcer* was assessed with the item, “Sometimes men have a sore or ulcer on or near their penis. During the last 12 months, have you had a sore or ulcer on or near your penis?” (Yes/No), and *genital discharge*, “Sometimes men experience an abnormal discharge from their penis. During the last 12 months, have you had an abnormal discharge from your penis?” (Yes/No). History of two or more sexual partners in the past 12 months.

*Current tobacco use* was assessed from two items, “Frequency smokes cigarettes” and “Frequency currently uses other type of tobacco.” *Current alcohol use* (at least one day in the past month).

*Early sexual debut* (≤ 14 years) was defined from the question of age of first sex.

*Dietary measures: women’s dietary diversity score (WDDS-10)*

During a 24-hour food recall, ten food groups were evaluated:

(1) Grains (white/pale starchy roots, tubers, and plantains), (2) Pulses (beans, peas, and lentils), (3) Nuts and seeds, (4) Dairy (milk, cheese, yogurt, other milk products), (5) Flesh foods (meat, fish, poultry, and liver/organ meats), (6) Eggs, (7) Dark green leafy vegetables, (8) Vitamin A rich fruits and vegetables, (9) Other vegetables and (10) Other fruits (scores 0–10) [34, 38].

Among women of reproductive age, the WDDS-10’s minimum food group consumption threshold of five out of the ten food groups is a reliable indicator of adequate dietary micronutrient intake [39].

## Psychosocial and environmental stressors

*Currently pregnant*,* has living children and pregnancy losses* (“How many miscarriages, abortions, and stillbirths have you had?”).

*Sons and/or daughters* who had died was measured from the questions, “Sons who have died” and “Daughters who have died.”

The following two questions were used to evaluate issues with *health care access*: “Is it a big problem in seeking medical advice or treatment for themselves when they are sick, 1) distance to the health facility, and 2) getting money for advice or treatment.”

*Use of solid cooking fuel* was assessed with the question what type of cooking fuel is used (ranging from 1 = electricity to 17 = sawdust) [34].

### Statistical analysis

To evaluate variations in percentages, chi-square statistics were employed. To estimate associations with PSB, MDD, and GAD, logistic regression was used, both unadjusted and adjusted (with variables significant in unadjusted analysis). Missing values were eliminated, and *P* < 0.05 was considered significant. The STATA software version 18.0 was utilized to conduct statistical analyses, considering the intricate study design. When using the Variance Inflation Factor (VIF) to check for collinearity, none was discovered.

## Results

The sample included 3,109 female adolescents (15–19 years), and 1,439 male adolescents (15–19 years). Tables 1 and 2 describe the sample characteristics and the distribution of, suicidal behaviour, MDD and GAD for girls and boys respectively. Among girls the prevalence of PSB was 4.3% (attempt 1.0%, plan 1.9% and/or ideation 3.6%) and among boys 2.5% (attempt 0.3%, plan 0.7% and/or ideation 2.4%). Among girls and boys, the prevalence of MDD (≥ 8 scores) was 15.5% and 3.7%, respectively, and the prevalence of GAD (≥ 5 scores) was 25.0% and 10.3%, respectively. Using MDD (≥ 10 scores), the prevalence was 8.6% among girls and 2.1% among boys, and using GAD (≥ 10 scores), the prevalence was 8.4% among girls and 1.6% among boys (see Tables 1 and 2).

**Table 1:** Sample characteristics and distribution of suicidal behaviour, major depressive disorder (MDD) and generalized anxiety disorder (GAD), girls, aged 15–19 years, demographic and Health Survey, Mozambique, 2022-23

Variables	Sample	Suicidal behaviour^a^	MDD (≥ 8 scores)	GAD (≥ 5 scores)
	N (%)	N (%)	N (%)	N (%)
**Sociodemographic factors**				
All	3109	128 (4.3)	409 (15.5)^b^	689 (25.0)^c^
Age (in years) 15–17 18–19	1760 (56.1) 1349 (43.9)	67 (3.7) 61 (5.0)	208 (14.5) 201 (16.8)	347 (22.5) 342 (28.2)
Marital status Not married/widowed/divorced Married/Cohabiting	2181 (68.8) 928 (31.2)	97 (4.4) 31 (3.9)	274 (15.0) 135 (16.6)	470 (23.9) 219 (27.4)
Education (> Primary)	1481 (41.3)	74 (5.8)	172 (13.4)	325 (23.4)
Wealth status (Poor/poorest)	788 (32.3)	21 (2.9)	135 (19.7)	204 (30.9)
Currently working	595 (16.2)	22 (3.8)	79 (14.4)	118 (21.9)
Urban residence (vs. rural)	1375 (41.5)	78 (6.0)	201 (15.8)	342 (25.6)
**Health status and behaviour**				
Self-rated health status (poor)	19 (0.3)	0 (0.0)	5 (28.5)	6 (32.6)
History of STI in the past 12 months	92 (2.7)	5 (6.2)	15 (13.3)	34 (35.9)
HIV positive	20 (0.5)	1 (6.2)	2 (18.5)	4 (33.5)
Tobacco use	32 (1.3)	0 (0.0)	8 (20.4)	10 (30.6)
Current alcohol use	112 (2.7)	8 (7.3)	11 (9.5)	23 (18.6)
Early sexual debut (≤ 14 years)	579 (17.5)	26 (4.3)	92 (18.5)	143 (30.0)
Dietary diversity (< 5)	2438 (79.8)	88 (3.8)	322 (16.2)	542 (25.4)
**Psychosocial and environmental stressors**				
Currently pregnant	225 (7.5)	8 (4.2)	46 (25.3)	66 (36.9)
Has living children	829 (27.1)	36 (4.4)	115 (16.5)	189 (26.8)
Pregnancy loss	116 (3.4)	5 (3.7)	14 (13.2)	28 (25.2)
Sons and/or daughters died	66 (2.4)	3 (3.0)	14 (28.8)	19 (41.0)
Big problem with distance to health facility	1080 (38.1)	30 (3.3)	139 (17.6)	247 (29.2)
Big problem to get money for medical treatment	885 (31.1)	29 (4.2)	178 (26.4)	267 (37.0)
Use of solid cooking fuel	2743 (89.8)	94 (3.7)	369 (16.1)	601 (30.9)

^a^Suicidal ideation (3.6%), suicide plan (1.9%) and/or suicide attempt (1.0%) in the past 12 months, ^b^PHQ-9 ≥ 10 scores: 8.6%; ^c^GAD-7 ≥ 10 scores: 8.4%); STI = Sexually Transmitted Infection; % are weighted

**Table 2:** Sample characteristics and distribution of suicidal behaviour, major depressive disorder (MDD) and generalized anxiety disorder (GAD), boys, aged 15–19 years, demographic and Health Survey, Mozambique, 2022-23

Variables	Sample	Suicidal behaviour^a^	MDD (≥ 8 scores)	GAD (≥ 5 scores)
	N (%)	N (%)	N (%)	N (%)
**Sociodemographic factors**				
All	1439	38 (2.5)	57 (3.7)^b^	153 (10.3)^c^
Age (in years) 15–17 18–19	577 (39.7) 862 (60.3)	15 (2.7) 23 (2.5)	16 (3.1) 41 (4.2)	46 (8.8) 107 (11.4)
Marital status Not married/widowed/divorced Married/Cohabiting	1384 (95.4) 55 (4.6)	36 (2.5) 2 (3.4)	55 (3.6) 2 (5.3)	147 (10.0) 6 (17.6)
Education (> Primary)	746 (47.1)	26 (4.0)	36 (5.0)	98 (11.9)
Wealth status (Poor/poorest)	326 (30.2)	6 (1.2)	10 (2.0)	25 (7.3)
Currently working	772 (53.7)	18 (2.1)	26 (3.0)	81 (10.8)
Urban residence (vs. rural)	620 (41.5)	27 (4.6)	41 (6.6)	88 (13.7)
**Health status**				
Self-rated health status (poor)	28 (2.9)	0 (0.0)	2 (2.9)	4 (9.3)
History of STI in the past 12 months	49 (3.2)	0 (0.0)	2 (3.1)	10 (23.7)
HIV positive	4 (0.1)	0 (0.0)	0 (0.0)	0 (0.0)
Genital sore	39 (2.5)	1 (2.4)	5 (13.2)	10 (26.7)
Genital discharge	52 (3.4)	3 (6.2)	5 (8.4)	11 (24.2)
**Stress and health risk behaviour**				
Has living children	47 (3.1)	0 (0.0)	5 (14.7)	9 (20.6)
Tobacco use	9 (0.6)	0 (0.0)	0 (0.0)	1 (8.2)
Current alcohol use	176 (10.9)	7 (5.2)	11 (8.0)	31 (20.6)
Early sexual debut (≤ 14 years)	316 (19.1)	8 (3.1)	18 (7.2)	44 (17.6)
Multiple sexual partners in the past 12 months	320 (19.3)	9 (2.7)	16 (4.7)	40 (12.4)

^a^Suicidal ideation (2.4%), suicide plan (0.7%) and/or suicide attempt (0.3%) in the past 12 months; ^b^PHQ-9 ≥ 10 scores: 2.1%; ^c^GAD-7 ≥ 10 scores: 1.6%); STI = Sexually Transmitted Infection; % are weighted

## Associations with past 12-month suicidal behaviour

In adjusted logistic regression analysis, among girls, GAD (Adjusted Odds Ratio-AOR: 3.12, 95% Confidence Interval-CI: 1.75–5.58) was positively and solid fuel use (AOR: 0.50, 95% CI: 0.29–0.85) was negatively associated with PSB, while among boys MDD (AOR: 7.89, 95% CI: 2.39–24.01) and urban residence (AOR: 2.52, 95% CI: 1.08–5.87) were positively associated with PSB (see Tables 3 and 4).

**Table 3:** Associations with suicidal behaviour among girls

Variables	Crude Odds Ratio (95% CI)	Adjusted Odds Ratio (95% CI)
**Sociodemographic factors**		
Age (in years) 15–17 18–19	1 (Reference) 1.37 (0.87 to 2.17)	–
Marital status Not married/widowed/divorced Married/Cohabiting	1 (Reference) 0.86 (0.52 to 1.42)	–
Education (> Primary)	1.91 (1.15 to 3.16)*	1.47 (0.86 to 2.54)
Wealth status (Poor/poorest)	0.57 (0.30 to 1.08)	–
Currently working	0.87 (0.49 to 1.53)	–
Urban residence (vs. rural)	2.01 (1.24 to 3.26)**	1.45 (0.87 to 2.41)
**Health status and behaviour**		
Self-rated health status (poor)	–	–
Depressive symptoms	2.48 (1.45 to 4.24)***	1.21 (0.60 to 2.48)
Anxiety symptoms	3.37 (2.19 to 5.19)***	3.12 (1.75 to 5.58)***
History of STI in the past 12 month	1.51 (0.52 to 4.36)	–
HIV positive	1.49 (0.19 to 11.51)	–
Tobacco use	–	–
Current alcohol use	1.82 (0.80 to 4.15)	–
Early sexual debut (≤ 14 years)	1.00 (0.55 to 1.82)	–
Dietary diversity (< 5)	0.63 (0.39 to 1.02)	–
**Psychosocial and environmental stressors**		
Currently pregnant	1.00 (0.36 to 2.77)	–
Has living children	1.06 (0.64 to 1.75)	–
Pregnancy loss	0.85 (0.30 to 2.43)	–
Sons and/or daughters died	0.70 (0.19 to 2.51)	–
Big problem with distance to health facility	0.68 (0.41 to 1.13)	–
Big problem to get money for medical treatment	0.97 (0.59 to 1.60)	–
Use of solid cooking fuel	0.39 (0.23 to 0.65)***	0.50 (0.29 to 0.85)**

CI = Confidence Interval; * *p*-value < 0.05; ** *p*-value < 0.01, *** *p*-value < 0.001; STI = Sexually Transmitted Infection

**Table 4:** Associations with suicidal behaviour among boys

Variables	Crude Odds Ratio (95% CI)	Adjusted Odds Ratio (95% CI)
**Sociodemographic factors**		
Age (in years) 15–17 18–19	1 (Reference) 0.93 (0.45 to 1.95)	–
Marital status Not married/widowed/divorced Married/Cohabiting	1 (Reference) 1.35 (0.31 to 5.87)	–
Education (> Primary)	3.22 (1.45 to 7.15)**	1.94 (0.81 to 4.65)
Wealth status (Poor/poorest)	0.38 (0.14 to 1.05)	–
Currently working	0.69 (0.33 to 1.43)	–
Urban residence (vs. rural)	4.28 (2.00 to 9.16)***	2.52 (1.08 to 5.87)*
**Health status**		
Self-rated health status (poor)	–	
MDD	12. 43 (5.24 to 29.50)***	7.89 (2.39 to 24.01)***
GAD	3.56 (1.53 to 8.29)***	1.38 (0.44 to 4.34)
History of STI in the past 12 month	–	–
HIV positive	–	–
Genital sore	0.93(0.13 to 6.49)	–
Genital discharge	2.67 (0.78 to 9.11)	–
**Stress and health risk behaviour**		
Has living children	–	–
Tobacco use	–	–
Current alcohol use	2.43 (0.87 to 6.77)	–
Early sexual debut (≤ 14 years)	1.30 (0.57 to 2.97)	–
Multiple sexual partners in the past 12 months	1.06 (0.47 to 2.42)	–

CI = Confidence Interval; * *p*-value < 0.05; ** *p*-value < 0.01, *** *p*-value < 0.001; STI = Sexually Transmitted Infection

### Associations with major depressive disorder

Among female adolescents, currently being pregnant (AOR: 1.82, 95% CI: 1.18–2.78) and “big problem to get money for medical treatment” (AOR: 2.93, 95% CI: 2.22–3.86) were positively associated with MDD. While among male adolescents, urban residence (AOR: 3.98, 95% CI: 1.99–7.97), having a genital sore or ulcer (AOR: 4.14, 95% CI: 1.53–11.23), has living children (AOR: 5.35, 95% CI: 1.80–15.90), and early sexual debut (AOR: 2.28, 95% CI: 1.23–4.24) were positively associated with MDD (see Tables 5 and 6).

**Table 5:** Associations with major depressive disorder among girls

Variables	Crude Odds Ratio (95% CI)	Adjusted Odds Ratio (95% CI)
**Sociodemographic factors**		
Age (in years) 15–17 18–19	1 (Reference) 1.19 (0.93 to 1.53)	
Marital status Not married/widowed/divorced Married/Cohabiting	1 (Reference) 1.13 (0.82 to 1.57)	–
Education (> Primary)	0.75 (0.59 to 0.97)*	1.24 (0.89 to 1.74)
Wealth status (Poor/poorest)	1.57 (1.11 to 2.21)**	1.28 (0.85 to 1.95)
Currently working	0.90 (0.65 to 1.25)	–
Urban residence (vs. rural)	1.04 (0.73 to 1.46)	–
**Health status and behaviour**		
Self-rated health status (poor)	2.18 (0.59 to 8.03)	–
History of STI in the past 12 month	0.83 (0.42 to 1.66)	–
HIV positive	1.24 (0.27 to 5.64)	–
Tobacco use	1.41 (0.54 to 3.65)	–
Current alcohol use	0.56 (0.29 to 1.11)	–
Early sexual debut (≤ 14 years)	1.31 (0.90 to 1.89)	–
Dietary diversity (< 5)	1.32 (0.95 to 1.82)	–
**Psychosocial and environmental stressors**		
Currently pregnant	1.97 (1.28 to 3.04)**	1.82 (1.18 to 2.78)**
Has living children	1.11 (0.83 to 1.49)	–
Pregnancy loss	0.83 (0.42 to 1.63)	–
Sons and/or daughters died	2.26 (1.09 to 4.68)*	1.65 (0.79 to 3.45)
Big problem with distance to health facility	1.29 (0.89 to 1.87)	–
Big problem to get money for medical treatment	3.04 (2.31 to 4.01)***	2.93 (2.22 to 3.86)***
Use of solid cooking fuel	1.65 (1.04 to 2.61)*	1.42 (0.89 to 2.26)

CI = Confidence Interval; * *p*-value < 0.05; ** *p*-value < 0.01, *** *p*-value < 0.001;

**Table 6:** Associations with major depressive disorder among boys

Variables	Crude Odds Ratio (95% CI)	Adjusted Odds Ratio (95% CI)
**Sociodemographic factors**		
Age (in years) 15–17 18–19	1 (Reference) 1.38 (0.73 to 2.60)	–
Marital status Not married/widowed/divorced Married/Cohabiting	1 (Reference) 1.47 (0.28 to 7.78)	–
Education (> Primary)	2.00 (1.07 to 3.77)*	1.68 (0.97 to 3.21)
Wealth status (Poor/poorest)	0.43 (0.18 to 1.01)	–
Currently working	0.65 (0.35 to 1.20)	–
Urban residence (vs. rural)	4.13 (2.01 to 8.46)***	3.98 (1.99 to 7.97)***
**Health status**		
Self-rated health status (poor)	0.77 (0.16 to 3.64)	–
History of STI in the past 12 month	0.83 (0.19 to 3.63)	–
HIV positive	–	–
Genital sore or ulcer	4.20 (1.50 to 11.79)**	4.14 (1.53 to 11.23)**
Genital discharge	2.50 (0.87 to 7.19)	–
**Stress and health risk behaviour**		
Has living children	4.94 (1.65 to 14.76)**	5.35 (1.80 to 15.90)**
Tobacco use	–	–
Current alcohol use	2.65 (1.21 to 5.81)*	1.68 (0.76 to 3.72)
Early sexual debut (≤ 14 years)	2.62 (1.38 to 4.96)**	2.28 (1.23 to 4.24)**
Multiple sexual partners in the past 12 months	1.38 (0.66 to 2.89)	–

CI = Confidence Interval; * *p*-value < 0.05; ** *p*-value < 0.01, *** *p*-value < 0.001; STI = Sexually Transmitted Infection

### Associations with generalized anxiety disorder

Among female adolescents, poorer wealth status (AOR: 1.32, 95% CI: 1.01–1.72), currently pregnant (AOR: 1.59, 95% CI: 1.13–2.24) and “big problem to get money for medical treatment” (AOR: 2.58, 95% CI: 1.94–3.42) were positively associated with GAD, while among male adolescents, urban residence (AOR: 1.41, 95% CI: 1.17–2.79), current alcohol use (AOR: 2.04, 95% CI: 1.13–3.67), and early sexual debut (AOR: 2.04, 95% CI: 1.25–3.33) were positively associated with GAD (see Tables 7 and 8).

**Table 7:** Associations with generalized anxiety disorder among girls

Variables	Crude Odds Ratio (95% CI)	Adjusted Odds Ratio (95% CI)
**Sociodemographic factors**		
Age (in years) 15–17 18–19	1 (Reference) 1.35 (1.08 to 1.70)**	1 (Reference) 1.25 (0.99 to 1.57)
Marital status Not married/widowed/divorced Married/Cohabiting	1 (Reference) 1.20 (0.94 to 1.55)	–
Education (> Primary)	0.86 (0.70 to 1.06)	–
Wealth status (Poor/poorest)	1.57 (1.21 to 2.03)***	1.32 (1.01 to 1.72)*
Currently working	0.81 (0.61 to 1.08)	–
Urban residence (vs. rural)	1.05 (0.82 to 1.35)	–
**Health status and behaviour**		
Self-rated health status (poor)	1.45 (0.44 to 4.80)	–
History of STI in the past 12 month	1.71 (0.93 to 3.13)	–
HIV positive	1.52 (0.47 to 4.86)	–
Tobacco use	1.32 (0.53 to 3.30)	–
Current alcohol use	0.68 (0.41 to 1.13)	–
Early sexual debut (≤ 14 years)	1.36 (1.02 to 1.81)*	1.26 (0.94 to 1.70)
Dietary diversity (< 5)	1.14 (0.87 to 1.49)	–
**Psychosocial and environmental stressors**		
Currently pregnant	1.85 (1.34 to 2.53)***	1.59 (1.13 to 2.24)**
Has living children	1.14 (0.88 to 1.47)	–
Pregnancy loss	1.01 (0.60 to 1.69)	–
Sons and/or daughters died	2.12 (1.21 to 3.73)**	1.43 (0.82 to 2.49)
Big problem with distance to health facility	1.43 (1.09 to 1.86)**	0.77 (0.55 to 1.07)
Big problem to get money for medical treatment	2.41 (1.92 to 3.03)***	2.58 (1.94 to 3.42)***
Use of solid cooking fuel	1.04 (0.74 to 1.45)	–

CI = Confidence Interval; * *p*-value < 0.05; ** *p*-value < 0.01, *** *p*-value < 0.001;

**Table 8:** Associations with generalized anxiety disorder among boys

Variables	Crude Odds Ratio (95% CI)	Adjusted Odds Ratio (95% CI)
**Sociodemographic factors**		
Age (in years) 15–17 18–19	1 (Reference) 1.33 (0.84 to 2.11)	–
Marital status Not married/widowed/divorced Married/Cohabiting	1 (Reference) 1.93 (0.66 to 5.59)	–
Education (> Primary)	1.39 (0.92 to 2.10)	–
Wealth status (Poor/poorest)	0.59 (0.33 to 1.06)	–
Currently working	1.13 (0.73 to 1.73)	–
Urban residence (vs. rural)	1.83 (1.19 to 2.83)**	1.41 (1.17 to 2.79)**
**Health status**		
Self-rated health status (poor)	0.88 (0.23 to 3.39)	–
History of STI in the past 12 month	2.83 (1.14 to 7.06)*	1.59 (0.55 to 4.58)
HIV positive	–	
Genital sore or ulcer	3.31 (1.38 to 7.95)**	1.82 (0.62 to 5.33)
Genital discharge	2.92 (1.23 to 6.93)*	1.42 (0.52 to 3.81)
**Stress and health risk behaviour**		
Has living children	2.34 (0.94 to 5.84)	–
Tobacco use	0.77 (0.09 to 6.35)	–
Current alcohol use	2,60 (1.51 to 4.46)***	2.04 (1.13 to 3.67)*
Early sexual debut (≤ 14 years)	2.27 (1.45 to 3.55)***	2.04 (1.25 to 3.33)**
Multiple sexual partners in the past 12 months	1.30 (0.81 to 2.09)	–

CI = Confidence Interval; * *p*-value < 0.05; ** *p*-value < 0.01, *** *p*-value < 0.001; STI = Sexually Transmitted Infection

### Utilization of mental health care

Among female adolescents with PSB, MDD and/or GAD (*n* = 804), 12 (1.1%) took medicine prescribed by a doctor or other healthcare worker for depression or anxiety or any other mental problems in the past two weeks. Among male adolescents who had MDD and/or GAD (*n* = 172), none took mental medical care for depression or anxiety in the past 2 weeks, while among male adolescents who had no MDD nor GAD two (0.1%) took medical mental care in the past 2 weeks.

Furthermore, among girls who reported experiencing any GAD-7 and/or PHQ-9 symptoms (*n* = 1871), 121 (5.0%) ever tried to seek help consisting of other family member (54.4%), friend (37.3%), religious leader (7.5%), doctor/medical personnel (9.6%), current/former spouse/partner (5.5%), neighbour (1.4%), community health worker/fieldworker (0.0%), social worker (0.4%), social service organization (0.0%), and other (0.2%).

Furthermore, among boys who reported experiencing any GAD-7 and/or PHQ-9 symptoms (*n* = 892), 174 (16.5%) ever tried to seek help consisting of other family member (61.4%), friend (49.5%), religious leader (4.1%), doctor/medical personnel (3.0%), current/former spouse/partner (2.7%), neighbour (4.3%), community health worker/fieldworker (0.0%), social worker (0.0%), social service organization (0.8%), and other (0.9%).

## Discussion

In this nationally representative community-based sample of female adolescents (15–19 years) and male adolescents (15–19 years) in Mozambique, the prevalence of PSB (4.3% in girls and 2.5% in boys) (1.0% and 0.3% attempt, 1.9% and 0.7% plans and 3.6% and 2.4% ideation, in girls and boys, respectively) in 2022/23 seems to be much lower than in previous adolescent school surveys in Mozambique (18.0% suicide attempt in past 12 months [4], and 17.7% suicidal ideation in the past 12 months in 2015 [5] and 16.4% and 11.3% past month suicidal ideation and planning, respectively, in 2019 [6], and lower than among in-school adolescents in 40 LMICs (17.2% past 12 months suicide attempt) [7]. However, lower rates of PSB (4.0%) were found based on household interview-surveys among adolescents in eight urban and rural sites in six different Africa countries in 2015 to 2017 [8]. In the same study attending school was not significantly associated with PSB in four countries and in Burkina Faso attending school was inversely associated with PSB [8]. Similarly, in an interview-administered household survey among adolescents in Kenya low rates of suicidal behaviour (4.6% ideation, 2.4% plan and 1.0% attempt) in the past 12 months were found [40]. It is possible that due to greater anonymity in a self-administered school survey than in an interview-administered household survey, higher rates of PSB were found in school than in household surveys. Similar results were found in a study among adults showing that higher rates of suicidal ideation and suicide attempt were found with more anonymous assessment measures compared to interview-administered measures [41], and among adolescents in a previous study in Malawi showed a lower prevalence in sexual behaviour (more sensitive topic) in the interviewer-administered survey than in the self-administered survey [42]. Furthermore, it is possible that there has been a decline of PSB in Mozambique over time. In fact, from 2009 to 2017, the pooled prevalence of suicide attempts in the LMICs decreased significantly from 17.6% in the first survey to 13.8% in the second survey, including in Africa (in Benin, from 28.7% in 2009 to 12.0% in 2016) [43], according to the results of a trend analysis on suicide attempts among school adolescents in 12 LIMC. Some societal changes in Mozambique may have influenced the lower prevalence rate of PSB. For example, youth (15–24 years) unemployment decreased from 8.3% in 2020 to 7.6% in 2023 in Mozambique [44].

We found a prevalence of current MDD (15.5%, 8 scores cut-off and 8.6%, 10 scores cut-off among girls and 3.7%, 8 scores cut-off and 2.1%, 10 scores cut-off among boys) and GAD (25.0%, 5 scores cut-off and 8.4%, 10 scores cut-off among girls and 10.3%, 5 scores cut-off and 1.6%, 10 scores cut-off among boys) in Mozambique, which in terms of females seemed to be similar to among adolescents and youth seeking psychological services in Mozambique (7.3% had PHQ-9, ≥ 11 scores), 9% had GAD-7, ≥ 10 scores) [13], and among school adolescents in Maputo (17.7% GAD, and 8.5% MDD) [11]. Rates of MDD and GAD in this study seemed to be also lower than in two local studies among adolescents in South Africa (32.1% PHQ-9: ≥10 scores and 17.9% GAD-7: ≥10 scores) [14]; and 33.5% PHQ-A (8 cut off) and 20.9% GAD-7 (cut off 6) [15], and among sub-Saharan adolescents the median point prevalence in the general population were 26.9% for depression, and 29.8% for anxiety disorders [12].

The survey showed that among girls, GAD was positively and solid fuel use was negatively associated with PSB, while among boys MDD and urban residence were positively associated with PSB. Among female adolescents, currently being pregnant and “big problem to get money for medical treatment” increased the odds of MDD. While among male adolescents, urban residence, having a genital sore or ulcer, has living children, and early sexual debut were positively associated with MDD. Urban residence, current alcohol use, and early sexual debut were positively associated with GAD in male adolescents, while poorer wealth status, being pregnant, and having a “big problem to get money for medical treatment” were positively associated with GAD in female adolescents.

Consistent with some research [7, 8, 13, 21], female adolescents had a higher rate of PSB, MDD and GAD than male adolescents. This finding could be because female adolescents experience specific pubertal changes and estradiol levels [45] and are more vulnerable to sexual violence impacting negatively on mental health [33, 46], and male adolescents are more likely to have strong peer support, which may be protective against poor mental health [47, 48]. Furthermore, gender-specific norms, social expectations and experiences may account for gender differences in PSB, MDD and GAD [49, 50]. For instance, males and females suffer negative childhood experiences and dysfunctional households in various ways. Boys tend to externalize these events via masculine norms, leading to worse behavioural regulation, aggressiveness and risk taking, whereas girls tend to internalize them via feminine norms, leading to mental health problems [49–51].

Poorer wealth status was only among female adolescents associated with GAD but not with MDD and PSB, whereas higher wealth status was linked to both MDD and GAD in a study conducted among female adolescents in Nepal [22]. Some previous research identified that older adolescents [22] was associated with MDD, and younger adolescent age was associated with GAD [25], while our results did not find statistically significant age differences in terms of PSB, MDD and GAD. This finding may be related to the fact that we only included 15-19-year-olds in this study and did not consider younger adolescents. Strikingly, among boys but not girls, urban residence increased the odds of PSB, MDD and GAD in this study. It is possible that rural boys less likely report PSB, MDD and GAD than urban boys or that male adolescents living in urban areas are confronted with more stresses and consequently develop more mental issues than male adolescents residing in rural areas [52].

In terms of health status factors, this study confirmed some earlier research [23, 32] that a male adolescent’s history of genital sores or ulcers was linked to MDDs and/or GADs. Psychosocial effects like embarrassment, depression, and anxiety can arise from a history of genital sores or ulcers [53]. While some previous studies [13, 23, 33] found an association between history of STI, HIV positive and poor mental health, while this study did not find any significant associations. The latter finding may be related to the low self-reported HIV positive status (0.5% among females and 0.1% among males) in this study. Consistent with previous study among adolescents in Africa [6, 8, 18], we found that among boys MDD and among girls GAD were associated with PSB. Furthermore, there was a strong correlation observed between the scores for GAD and MDD (analysis not shown), indicating the high comorbidity of the two disorders in this population [10, 25].

Stressors included current pregnancy, limited access to healthcare, and having children, which were positively correlated with MDD and/or GAD in female adolescents and male adolescents, respectively. Adolescent mental health promotion may include adolescent mental health disorders (MDDs) and/or GADs because of psychosocial stress related to adolescent pregnancy and challenges in seeking medical attention for girls, as well as adolescent parenting and engaging in health-risk behaviors (alcohol use and early sexual debut) for boys. The negative effects of adolescent pregnancy may be reflected in the association between the current pregnancy and GAD and MDD [30]. A previous review found that the household use of solid biomass fuel was associated with MDD [31], while we found the use of solid cooking fuel was negatively associated with MDD in this study. This result may be related to the fact that is this study 90% used solid cooking fuel, and needs further research. Other health risk behaviours, such as tobacco use, having past 12-month multiple sexual partners and low dietary diversity, that were found significant in previous studies [24, 25, 28] showed no associations with MDD and/or GAD in this study.

Moreover, the study found that among female adolescents with PSB, MDD and/or GAD 1.1% took medicine medically prescribed for depression or anxiety or any other mental problems in the past 2 weeks, and among male adolescents who had MDD and/or GAD, none took medicine medically prescribed for depression or anxiety in the past 2 weeks. Main help seeking agents for mental symptoms in this study were family members, friends, religious leaders, and health care providers. There is evidence in Mozambique that patients are seeking treatment from traditional and/or faith healers who are providing psychosocial interventions that may might help in reducing MDD and/or GAD symptoms. The challenge to integrate local knowledge and healing practices remains unsolved as well how qualitative changes that are captured poorly by conventional rating scales might considered in this kind of studies [54, 55]. This finding shows the low utilization of mental health care, which is in line with previous research [56]. For example, among school adolescents with a mental disorder in Maputo only 7.3% had ever used specialized care, and 87.3% would seek help from primary care clinics if they thought they had a mental health problem [11]. To close this enormous mental health treatment gap in Mozambican adolescents “efforts to integrate mental health services within Mozambican primary care are warranted” [11].

Adding mental health services to primary care, following the implementation of a task-shifting strategy, Mozambique was able to reduce treatment gaps and expand mental health services to all districts of the country. The strategy was centered on the provision of services in primary care by midlevel professionals known as psychiatric technicians [57]. Muanido et al. [58] emphasize the implementation of common mental disorders screening and treatment integration into primary care, especially with general out-patients and with young people for depression. To enable continued expansion of mental health services in Mozambique, models, and decision-support instruments for the integration of mental healthcare with primary care practice are required [59]. In this effort, a cascade analysis tool (CAT) identifies the step within a cascade that could most improve the health care system and has been adapted to a mobile application for mental health outpatient services in Mozambique [60]. Furthermore, proactive psychosocial strategies by psychosocial support centers aimed at women, people living in cities, people with a history of genital sores or ulcers, people who are having difficulty accessing healthcare, people who are pregnant, people with children, and others should be put into practice with government support. Additionally, increased efforts in health promotion to lower risk behaviours, particularly early sexual debut, and alcohol use, may help prevent PSB, MDD, and GAD. Specifically, mental health interventions provided in the school setting can also be successful in lowering the prevalence of mental health issues in adolescents [61].

The Ministry of Health, Mozambique, has set priorities for 2014 to 2024, including the creating of a Mental Health Advocacy Forum do raise public opinion on the rights of people living with mental health challenges [62, 63]. Public campaigns have been conducted to promote mental health and fight the stigma around mental health. For instance, in September 2024, several activities around suicide prevention took place in schools, universities and mass media.

There is a growing cooperation among the Ministry of Health and traditional healers, religious leaders and community leaders about primary and secondary prevention of mental health, yet this effort is not systematic. Promising preventions campaigns such as “My Body belongs to me” aiming at addressing sexual abuse in public schools in the Cities of Maputo and Nampula, as it was developed by the Mozambican NGO “Reconstruindo a Esperança,” ARES, and the Mozambican Association for Psychology, in the years 2011–2013, fail to be integrated in the school curricula due lack of funding by the Ministry of Education. There are currently no psychologists employed by the Ministry of Education in schools to assist in promoting school children’s mental health. During the civil war, 1977–1992, the Ministry of Education had a successful programme that aided “Children in difficult circumstances”, due the military violence. Teachers and parents were trained on how to identify mental health symptoms and how to aid or to refer children and adolescents in need of mental health and psychosocial assistance [64, 65]. Another challenge in the Mental Health field in Mozambique is the promotion a permanent dialogue and cooperation between Mental Health professionals and Traditional Healers as it requires a shared understanding of the causes, ways to prevent and cure mental health disturbances. This dialogue requires the involvement of universities and psychological and medical professional organizations. There are some promising initiatives such as “Decolonizing Psychology” led by the Mozambican Circle of Psychanalysis in cooperation with the Instituto Superior Mutasa and the University of South Africa. Another initiative is a mental health and psychosocial project helping children and their families displaced by the military violence in northern Mozambique. This initiative is promoting dialogue and cooperation between mental health professionals from the International Childhood development Programmes, ICDP, and the Mozambican Association “Reconstruindo a Esperança” and the traditional healers and religious leaders in the city of Nacala.

### Study limitations and strengths

Standardized techniques and nationally representative samples—which included teenagers enrolled in and not—were employed in the study. However, because the study design is cross-sectional, we are unable to determine causal relationships. Some of the variables, such as the domestic violence module, body mass index and anemia, were only assessed in a survey sub-sample, and were therefore not included in the analysis. Furthermore, this survey did not evaluate some concepts related to mental distress, such as sleep and social support; these may be included in subsequent research. Other mental health conditions, such as substance use disorders [66, 67], and eating disorders [50], and other adverse child hood experiences [68, 69] that could impact on suicidal behaviour, anxiety, and depression in this adolescent population in Mozambique were not assessed, and should be included in future studies. Another limitation was that only older adolescents (15–19 years) and not younger adolescents (10–14 years) were included in this study, as some previous studies [22, 25] found differences between younger and older adolescents in relation to GAD and MDD. Therefore, future research should include the full age range of adolescents in the study. Data assessed were by interviewer-administered questionnaire, which may have led to social desirability bias, especially in sensitive topics (PSB, GAD and MDD) [41, 42]. Furthermore, longitudinal studies could provide insights into the development, specific moderating sociocultural factors, and progression of mental health problems over time [6].

## Conclusions

About 3% of adolescents had PSB, among girls one in five had MDD or GAD and among boys more than 5% MDD or GAD in Mozambique. Several factors associated with PSB, MDD and/or GAD were found among female and/or male adolescents, including female sex, poor wealth status, urban residence, currently pregnant, poor access to medical care, early sexual debut, alcohol use, having a genital sore or ulcer, and having living children, which can guide public health interventions to prevent PSB, MDD and/or GAD among adolescents in Mozambique. The findings can emphasize the need for comprehensive mental health interventions that consider both individual health behaviours and societal factors and recognizing and providing solutions to deal with the complex interplay of sociodemographic, psychosocial, and environmental factors that contribute to PSB, MDD and GAD.

## Data Availability

The data are available at the Demographic and Health Surveys Repository at https://dhsprogram.com/methodology/survey/survey-display-564.cfm.

## Abbreviations

DHS: Demographic and Health Survey
GAD: Generalised Anxiety Disorder
INE: The Instituto Nacional de Estatística
LMIC: Low- and middle-income country
MDD: Major Depressive Disorder
MINI-KID: Mini-International Neuropsychiatric Interview for Children and Adolescents
PSB: Past 12-month suicidal behaviour
STI: Sexually transmitted infection
WDDS-10: Women’s Dietary Diversity Score
